# Effect of *Apium graveolens* Extract Administration on the Pharmacokinetics of Captopril in the Plasma of Rats

**DOI:** 10.3390/scipharm86010006

**Published:** 2018-02-16

**Authors:** Siska Siska, Abdul Mun`im, Anton Bahtiar, Franciscus D. Suyatna

**Affiliations:** 1 Faculty of Pharmacy, Kampus UI Depok, Universitas Indonesia, West Java 16424, Indonesia; siska@uhamka.ac.id (S.S.); abdul.munim61@ui.ac.id (A.M.); anton.bahtiar@farmasi.ui.ac.id (A.B.); 2 Faculty of Pharmay and Science, Universitas Muhammadiyah Prof. Dr. HAMKA, East Jakarta 13460, Indonesia; 3 Departement of Pharmacology and Therapeutics, Faculty of Medicine, Universitas Indonesia, Central Jakarta 10430, Indonesia

**Keywords:** *Apium graveolens*, captopril, celery extracts, pharmacokinetics

## Abstract

*Apium graveolens* (celery) is an edible and traditionally medicinal plant that is used worldwide, among others for the treatment of hypertension. Combining celery with antihypertensive drugs can affect the pharmacodynamics and pharmacokinetics of the latter drugs. The aim of the study is to assess the effects of administrating the celery extract on captopril pharmacokinetics. Sprague-Dawley strain rats were divided into two groups (*n* = 6). Group I was given captopril (10 mg/kg Body Weight (BW)) orally, while Group II was pretreated with celery extract orally (40 mg/kg BW) an hour before administration of captopril. The blood samples were withdrawn at various intervals after drug administration. The captopril concentration was determined using liquid chromatography–mass spectrometry (LC-MS/MS) and from the blood data, the values of *K*_e_, *C*_max_, *T*_max_, *T*_1/2_, and area under the curve (*AUC*) were calculated. The results showed that oral administration of the celery extract increased *C*_max_ (38.67%), *T*_1/2_ (37.84%), and *AUC* (58.10%) and decreased *K*_e_ (27.45%) of captopril in Group II (celery + captopril) compared with Group I (captopril). In conclusion, celery extract can alter the pharmacokinetic of captopril when given in combination. The combination might be beneficial for the treatment of hypertension, as celery causes an increase in the plasma level of captopril, which can enhance its efficacy.

## 1. Introduction

Celery (*Apium graveolens*) is usually used in traditional medicine as a diuretic or anti-hypertensive agent. It contains chemical compounds such as apiin, apigenin, isoquercitrin, and sesquiterpene [1,2]. Celery has been sold as food supplement, so the pharmaceutical industries do not need to prove the efficacy of the herbs or determine the side effects or interactions between the products [3]. Most people believe that herbs are harmless plants but can affect body functions. In fact, in Indonesia, herbs are frequently used as traditional medicines for various diseases. Since herbs are widely available, people can easily use them alone or in combination with synthetic drugs. There is limited knowledge about herbs, and interactions may occur when herbs are taken together with synthetic drugs [3]. The mechanisms of drug interactions can be divided into several general categories, including pharmacokinetics (absorption, distribution, metabolism, and excretion of a drug) and pharmacodynamics (the combined pharmacological effects of a drug) [3]. Combining synthetic drugs with herb preparations may have a synergistic effect or can increase the rate of side effects [4]. 

Captopril is widely used as an antihypertensive agent and for heart failure. It is the first drug from the class of angiotensin converting enzyme inhibitors (ACEis), which works by preventing the conversion of angiotensin I into angiotensin II (potential vasoconstrictor and aldosterone secretion stimulant agent). ACE inhibitors prevent the degradation of bradykinin and stimulate the synthesis of compounds, including other vasodilators, such as prostaglandin E2 and prostacyclin. Decreased secretion of aldosterone causes sodium and water excretion as well as potassium retention. Subsequently, this causes a decline in blood pressure in patients with hypertension [5].

Captopril and celery have been used in Indonesia to treat hypertension [6]. Previous research has provided evidence that 73% of patients in public hospitals have used captopril as an antihypertensive drug [7]. Other research has shown that there was evidence that 71.4% of patients in the public community health center have combined celery herbs with captopril, finding that this combination is able to reduce blood pressure better than captopril alone [8]. Another study reported that celery juice has the potential to modulate cytochrome P450 activity and thus can interact with synthetic drugs [9]. Therefore, the exact mechanisms of the captopril–celery interaction are unknown. We aimed to study the interaction of celery with captopril to evaluate the pharmacokinetic interaction of captopril with celery using rat as the experimental animal model.

## 2. Materials and Methods

### 2.1. Experimental Animals

White male Sprague-Dawley rats used in this experiment were obtained from the Bogor Agricultural University (IPB), Bogor, West Java, Indonesia; weighing 200–250 g. The rats were housed at 25 ± 5 °C in a well-ventilated animal house under a 12:12 h light/dark cycle. The rats had free access to food and water. The study protocol has been approved by the Health Research Ethics Committee of the Faculty of Medicine, Universitas Indonesia, Central Jakarta, Indonesia. The reference number for approval was 666/UN2.F1/ETHICS/2016.

### 2.2. Preparation of Celery Extract and Physicochemical Evaluation

Fresh celery (*Apium graveolens*) was purchased from the local market. The plant was identified at The Research Center for Biology, Indonesian Institute of Sciences (LIPI), Cibinong, West Java, Indonesia; with the determination specimen number of 1777/IPH.1.01/if.07/VIII/2016. The identification of the plant was conducted to determine the classification of the plants [10]. Celery herbs were rinsed with running water. The small cut pieces of the sample were dried at room temperature or in the oven to prevent microbial fermentation and the degradation of metabolites as well as to minimize chemical reactions that were induced by ultraviolet rays from direct sunlight. After this, the dried celery powders were macerated using 50% ethanol for three days. The extract was separated by filtration using filter paper and was concentrated using rotary vacuum evaporator (Buchi, Darmstadt, Germany). The viscous extract was collected and stored at 4 °C before used [11]. 

The physico-chemical characteristics of the extract were determined according to methods described by WHO guidelines on the quality control methods for medicinal plant materials [12] and the standard for medicinal plant extract through the following quality parameters: water content, total ash content, loss on drying, and essential oil content [13]. 

### 2.3. Chromatography

The chromatography was performed on C18 column Acquity (Waters, Milford, CT, USA) (100 mm × 2.1 mm), with a particle size of 1.7 µm at a temperature of 40 °C. The gradient system of the mobile phase composition was a mixture of 0.1% formic acid and acetonitrile (60:40 *v*/*v*), with a flow rate of 0.3 mL/second. For mass detection, we used a Waters Xevo Triple Quadrupole (Waters) equipped with an electrospray ionization (ESI) source in positive ions in multiple reaction monitoring (MRM) mode.

### 2.4. Preparation of the Standard Solution: Calibration Standards and Quality Control Sample Preparation

A stock solution of captopril with a concentration of 100 µg/mL was prepared by dissolving 40 mg in 100 mL of water. A stock solution of propranolol (1 mg/mL) and apigenin (1 mg/mL) was prepared by dissolving 40 mg in 100 mL of methanol. Calibration standards and the Quality Control (QC) sample were prepared by diluting the stock solution with Sprague-Dawley rat plasma to form the calibration standards of captopril in the presence of apigenin (3, 6, 12, 25, 50, and 100 ng/mL) and QC sample (9, 40, and 80 ng/mL). A stock solution of 2,4-dibromo acetophenone with a concentration of 520 µg/mL was prepared by dissolving 52 mL in 100 mL of methanol. 

### 2.5. Preparation of Plasma Sample

A simple protein precipitation procedure was applied to clean up the plasma sample before use [14]. A total of 180 µL of plasma containing the specific concentration of captopril and apigenin was added to 20 µL of 2,4-dibromo acetophenone working solution and 5% ammonia solution. After this, the solution was vortexed for 30 s and stored at 25 °C for 30 min, before adding 20 µL of 15% formic acid, propranolol (1 µL/mL), and 600 µL of acetonitrile. The solution was vortexed for 30 s and centrifuged at 12,000 rpm for 5 min. The supernatant was transferred to an autosampler vial, before 5 µL was injected into the liquid chromatography–mass spectrometry (LC-MS/MS) system. 

### 2.6. Pharmacokinetic Study

Twelve rats were divided into two groups: Group I received a single captopril at a dose of 10 mg/kg orally, while Group II was pretreated with the celery extracts orally (40 mg/kg) an hour before the administration of captopril. Serial blood samples (0.5 mL per sample) were collected before dosing and after 10, 20, 30, 45, 60, 120, 180, 300, and 420 min. The harvested plasma samples were treated in the pre-used plasma preparations mentioned before. The captopril concentration was determined using LC-MS/MS, and the values of elimination constant (*K*_e_), maximum concentration (*C*_max_), maximum time (*T*_max_), half-time (*T*_1/2_), and area under the curve (*AUC*) were calculated based on this blood data.

The data were represented in a plasma level–time curve, which was used to calculate the *AUC*_0-7h_ using the trapezoid rule. The *C*_max_ and *T*_max_ were obtained directly from the generated data. The *K*_e_ and *T*_1/2_ were determined from the semi-log plot of the data. The mean plasma concentration–time curve for captopril (10 mg/kg) alone and captopril + celery extract (40 mg/kg) was determined. The study was conducted for 7 h since the half-life of captopril is 1–2 h [15]. The results were analyzed statistically using the Student’s *T*-test. 

## 3. Results

### 3.1. Physico-Chemical Characteristics

The physicochemical characteristics, such as weight loss after drying, ash values, water content, and essential oil content, are given in Table 1.

### 3.2. Optimization of LC-MS/MS Parameters

For mass detection, we used Water Xevo TQD (Waters) equipped with electrospray ionization (ESI) source in positive ions in multiple reaction monitoring (MRM) mode. The following operational parameters of the ion cone and collision energies are presented in Table 2. Captopril was detected at an *m/z* of 271.13 > 153.07, while propranolol was detected at an *m/z* of 260 > 183.17, which was used as an internal standard. 

### 3.3. Calibration Curve and Lower Limit of Quantification (LLOQ)

The curves of calibration were found to be linear, over the concentration range of 3–100 ng/mL and with a linearity of 0.9961, and the lower limit of quantification (LLOQ) was 3 ng/mL. The precision value (%CV) of within-run analysis was 6.91–8.37%, which is less than 20% (Table 3).

### 3.4. Pharmacokinetics

**Figure 1:**
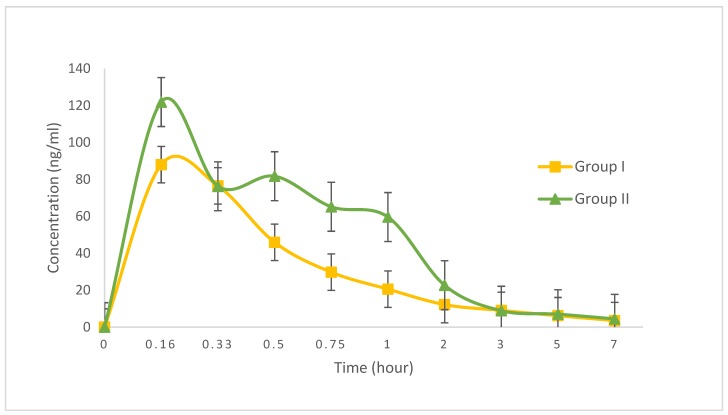
Pharmacokinetics of captopril. Plasma captopril levels were measured with LC-MS/MS. Symbols represent the mean concentration ± standard error of the mean (SEM). Group I (*n* = 6) was given a single dose of captopril (10 mg/kg, orally), while Group II (*n* = 6) was given captopril (10 mg/kg) with celery extract (40 mg/kg, orally).

The pharmacokinetics of the combined captopril and celery extract were subsequently examined in Sprague-Dawley rats after oral administration. The pharmacokinetic parameters are listed in Table 4 and Figure 1. The *C*_max_ and *AUC*_total_ was reached in Group II compared with Group I, although there were no significant differences (*p* > 0.05).

## 4. Discussion

The physico-chemical characteristics of celery extract determine their quality. Water content or moisture is sufficient to facilitate the activation of enzymes and the proliferation of microorganisms. These are inevitable components of crude medicines and must be eliminated as much as possible. Ash values are useful in determining the authenticity and purity of a drug as well as its critical quantitative standards. The total ash value of the extract provides an indication of earthy matter or mineral composition as well as the impurities present. The quality of herbal medicines can be affected by many factors, such as light exposure, temperature, water availability, the amount of nutrients, the period and time of collection, the methods by which the medicine is collected, dried, packed, stored, and transported, age, and which part of the plant is collected [12,13]. 

Captopril is an unstable compound that undergoes oxidation. The degradation product is a dimer that also binds to endogenous compounds (cysteine and glutathione) [15]. A derivatizing agent, 2,4-dibromo acetophenone, was added to improve the stability of the compound, which prevents captopril from binding to plasma constituents and is also a chemical stabilizer [15].

The precision and accuracy of captopril in the presence of apigenin (a marker from celery extract) were calculated by our within-run variation of QC samples at four different concentrations with five replicates. The precision (%CV) value from the within-run analysis is 6.91–8.37%, while the accuracy (%diff) of captopril is less than 20%. The accuracy and precision values indicate the adequate reliability and reproducibility of the method within an analytical range [16]. 

The pharmacokinetic parameter values of captopril were calculated (Table 4). The celery extract was administered one hour before captopril in order to ensure that there was no interaction with the absorption of captopril. The pharmacokinetic interaction appears to occur at the cytochrome P450 level [17,18]. 

The calculation of pharmacokinetic parameters showed that the concomitant use of captopril and celery extract increased the plasma levels (*C*_max_) by 38.67% and *AUC*_total_, of captopril, although there were no significant differences (*p* > 0.05). The time needed to reach the peak (*T*_max_) plasma concentration of captopril occurred at the same time in both groups. There was prolonged elimination half-life (*T*_1/2_) of captopril in the presence of celery from 2.93 to 4.03. Although there were no significant differences (*p* > 0.05), the upward trend of *C*_max_ and *AUC* of captopril combined with celery extract in our study suggests that celery extract was a potent inhibitor of cytochrome P450, which is responsible for captopril metabolization [9].

Therefore, if captopril is taken along with celery, this will inhibit the metabolism of captopril. A previous study showed that captopril has pharmacodynamical interactions when combined with garlic, which has the synergetic effect of preventing the damage caused by isoproterenol in rats [19].

The mechanism of action for many herbs has not been determined and the exact mechanisms of drug–herb interaction are also unknown [20]. To our knowledge, this is the first report showing the possible pharmacokinetics interaction of celery extract when combined with captopril.

## 5. Conclusions

The administration of celery extracts, when given in combination with captopril, can increase the bioavailability of captopril which is probably caused by inhibition of the cytochrome P450 responsible for captopril catabolism

## Figures and Tables

**Table 1:** Physicochemical characteristics of celery extract.

Parameter	Celery Extract
Ash values (%) *w*/*w*	6.70%
Water content (%) *v*/*w*	8.89%
Essential oil (%) *v*/*w*	3.34%
Loss on drying (%) *w*/*w*	4.87%

**Table 2:** The result of optimization detection of liquid chromatography–mass spectrometry (LC-MS/MS).

Compound	Parent (*m*/*z*)	Daughter (*m*/*z*)	Cone (V)	Collison (V)	Area
Captopril	415	216.16	35	17	3.05 x 10^5^
Propranolol	260	183.17	42	17	5.59 x 10^6^
Apigenin	271.13	153.07	61	31	7.03 x 10^6^

**Table 3:** Accuracy and precision of captopril in presence of apigenin.

Concentration (ng/mL)	Mean Measured Concentration (ng/mL) ± Standard Deviation	(%CV)	(%diff)
3	3.13 ± 0.25	7.91	4.46
9	8.46 ± 0.71	8.37	−5.82
40	39.36 ± 2.93	7.46	−1.41
80	77.37 ± 5.34	6.91	−3.10

**Table 4:** Pharmacokinetic parameters of captopril.

Pharmacokinetic Parameters	Group I (*n* = 6) (single captopril)	Group II (*n* = 6) (captopril + celery extract)
*C*_max_ (ng/mL)	100.63 ± 28.62	127.86 ± 29.30
*T*_max_ (hour)	0.16	0.16
*K*_e_ (hour^−1^)	0.2391 ± 0.08	0.2162 ± 0.07
*T*_1/2_ (hour)	1.8578 ± 3.67	4.7029 ± 1.05
*AUC*_total_	99.11 ± 13.05	158.28 ± 25.64

Values are mean ± SEM, *p* > 0.05 when compared to captopril alone. *AUC*: area under the curve; *C*_max_: maximum concentration; *T*_max_: maximum time; *K*_e_: elimination constant; *T*_1/2_: half-time.
